# Mitochondrial potassium channels: A novel calcitriol target

**DOI:** 10.1186/s11658-021-00299-0

**Published:** 2022-01-03

**Authors:** Anna M. Olszewska, Adam K. Sieradzan, Piotr Bednarczyk, Adam Szewczyk, Michał A. Żmijewski

**Affiliations:** 1 Department of Histology, Medical University of Gdańsk, 1a Dębinki, 80-211 Gdańsk, Poland; 2 Faculty of Chemistry, University of Gdańsk, Wita Stwosza 63, 80-308 Gdańsk, Poland; 3 Department of Physics and Biophysics, Warsaw University of Life Sciences-SGGW, Nowoursynowska 159, 02-776 Warsaw, Poland; 4 Laboratory of Intracellular Ion Channels, Nencki Institute of Experimental Biology, Polish Academy of Sciences, 02-093 Warsaw, Poland

**Keywords:** Calcitriol, Large-conductance calcium-regulated potassium channel, Mitochondria, Patch-clamp

## Abstract

**Background:**

Calcitriol (an active metabolite of vitamin D) modulates the expression of hundreds of human genes by activation of the vitamin D nuclear receptor (VDR). However, VDR-mediated transcriptional modulation does not fully explain various phenotypic effects of calcitriol. Recently a fast non-genomic response to vitamin D has been described, and it seems that mitochondria are one of the targets of calcitriol. These non-classical calcitriol targets open up a new area of research with potential clinical applications. The goal of our study was to ascertain whether calcitriol can modulate mitochondrial function through regulation of the potassium channels present in the inner mitochondrial membrane.

**Methods:**

The effects of calcitriol on the potassium ion current were measured using the patch-clamp method modified for the inner mitochondrial membrane. Molecular docking experiments were conducted in the Autodock4 program. Additionally, changes in gene expression were investigated by qPCR, and transcription factor binding sites were analyzed in the CiiiDER program.

**Results:**

For the first time, our results indicate that calcitriol directly affects the activity of the mitochondrial large-conductance Ca^2+^-regulated potassium channel (mitoBK_Ca_) from the human astrocytoma (U-87 MG) cell line but not the mitochondrial calcium-independent two-pore domain potassium channel (mitoTASK-3) from human keratinocytes (HaCaT). The open probability of the mitoBK_Ca_ channel in high calcium conditions decreased after calcitriol treatment and the opposite effect was observed in low calcium conditions. Moreover, using the AutoDock4 program we predicted the binding poses of calcitriol to the calcium-bound BK_Ca_ channel and identified amino acids interacting with the calcitriol molecule. Additionally, we found that calcitriol influences the expression of genes encoding potassium channels. Such a dual, genomic and non-genomic action explains the pleiotropic activity of calcitriol.

**Conclusions:**

Calcitriol can regulate the mitochondrial large-conductance calcium-regulated potassium channel. Our data open a new chapter in the study of non-genomic responses to vitamin D with potential implications for mitochondrial bioenergetics and cytoprotective mechanisms.

**Supplementary Information:**

The online version contains supplementary material available at 10.1186/s11658-021-00299-0.

## Background

The bioactive vitamin D metabolite calcitriol (1,25(OH)_2_D_3_) is a naturally occurring hormone with pleiotropic activity and the key regulator of calcium-phosphate homeostasis. Vitamin D is formed in a UVB-dependent, non-enzymatic reaction from 7-dehydrocholesterol in the upper most layer of the skin—the epidermis, but its full biological activity requires two subsequent hydroxylations by CYP2R1 in the liver and CYP27B1 in kidneys. In addition to the long-known important role in regulating the level of calcium in the body, calcitriol is implicated in the modulation of cell differentiation, proliferation, and also in the prevention of various diseases, including cancer [1–4]. Calcitriol acts via the vitamin D nuclear receptor (VDR) activating the classic genomic pathway, but rapid non-genomic responses to vitamin D have recently been reported (for a recent review see Zmijewski et al. [5]). VDR-independent vitamin D signaling also occurs via membrane-bound proteins, such as protein disulfide isomerase family A member 3, also known as endoplasmic reticulum resident protein 57 (ERp57), and membrane-associated rapid response to steroid (1,25D_3_-MARRS) [6]. Alternatively, calcitriol can act in a non-genomic way by binding to an alternative ligand-binding pocket to the classic nuclear VDR [7]. It is worth emphasizing that activity of other nuclear receptors could be modulated by vitamin D derivatives, such as retinoic acid-related orphan receptors (RORs) by 20-hydroxyvitamin D_3_ and 20,23-dihydroxyvitamin D_3_ [8], liver X receptors (LXR) by the derivatives of lumisterol and vitamin D [9], and aryl hydrocarbon receptor (AhR) by 20,23-dihydroxyvitamin D_3_ [10]. Also, it seems interesting that the VDR is translocated into the mitochondria of some cell types. Description of non-genomic pathways was used to explain particular effects which could not be attributed to the activation of VDR in the nucleus [5].

In the inner mitochondrial membrane, there are heme-containing enzymes involved in the metabolism of vitamin D belonging to the cytochrome P450 family, such as CYP27A1, CYP27B1, CYP24A1, and CYP11A1. The last of the mentioned cytochromes, CYP11A1, initiates novel pathways of vitamin D metabolism, which was observed in the placenta [11], adrenal glands [12], and epidermal keratinocytes [11, 13]. Recently, it was found that the products of CYP11A1 activity may be further modified by CYP27B1 [14]. As mentioned above, steroid receptors, including VDR, besides being in the nucleus and cytoplasm, can be localized in mitochondria from human platelets [15], megakaryocytes [16], keratinocytes [17], and fibroblasts [18]. In contrast to ligand-dependent nuclear VDR, its distribution in mitochondria seems to be ligand-independent. Due to no obvious N-terminal import sequence in the mitochondrial form of VDR, it is suggested that these receptors are not transported through translocase of the outer and inner mitochondrial membrane (TOM/TIM translocase), but through the permeability transition pore (PTP) dependent pathway [17]. It was proved that VDR interacts with PTP, the voltage-dependent anion-selective channel (VDAC), and steroidogenic acute regulatory protein (StAR) protein, which regulates cholesterol transfer within mitochondria [17]. The role of VDR in mitochondria is still under debate. Interestingly, mitochondrial localization of VDR is a common feature in cancer proliferating cell lines rather than fully differentiated ones [19]. It has been reported that mitochondrial VDR supports cancer cell metabolism and redirects tricarboxylic acid cycle (TCA) intermediates towards biosynthesis. Additionally, VDR knockdown increases mitochondrial membrane potential, enhances ROS production [20], and increases expression of components of the respiratory chain, such as cyclooxygenase 2 (COX2), cyclooxygenase 4 (COX4), the ATP synthase membrane subunit (6MT-ATP6) encoded by mitochondrial genome and ATP synthase subunit beta (ATP5B) [20]. However, it should be taken into account that long-term VDR silencing leads to loss of mitochondrial integrity and cellular damage [19]. Treatment of keratinocytes with vitamin D downregulates the protein level of the subunits of the respiratory chain [20], affects mitochondrial membrane potential, and responds to oxidative stress [21]. Interestingly, in silico analysis points to direct transcriptional control of mitochondrial encoded proteins via vitamin D response elements (VDRE) presented in the displacement loop (D-loop) of the mitochondrial genome [22].

The regulation of the plasma membrane ion channels by calcitriol has been reported mainly at the gene expression and protein level, and there are only sparse electrophysiological data concerning direct channel activity. Transient receptor potential cation channels (TRPV) are best described in the context of calcitriol regulation due to their contribution to cell proliferation and regulation of calcium influx. In general, calcitriol treatment increases the expression level of TRPV1 [23, 24], TRPV5 [25], and TRPV6 [26]. Interestingly, the opposite effect has been observed in studies of potassium channels Kv10.1 [27, 28] and TASK-1 [29]. In the case of electrophysiological experiments, calcitriol at physiologically relevant concentrations (100 nM) acts as a mild agonist of the transient receptor potential cation channel TRPV1, but at higher concentrations of the TRPV1-specific agonist capsaicin acts as an inhibitor of the channel [23, 30]. Molecular docking studies have shown that calcitriol interacts directly with the channel protein by binding to the vanilloid binding pocket as capsaicin [23]. Nevertheless, until now, there have been no electrophysiological studies conducted at the single-channel level on mitochondrial potassium channels.

A large amount of evidence has implicated mitochondrial potassium channels as potentially playing a significant role in cell-protective strategies. To date, eight different potassium channels have been identified in the inner mitochondrial membrane: mitochondrial small-conductance calcium-regulated potassium channel (mitoSK) [31], mitochondrial intermediate-conductance calcium-regulated potassium channel (mitoIK) [32], mitoBK_Ca_ [33], mitoTASK-3 [34], mitochondrial voltage-dependent potassium channel type 1.3 (mitoKv1.3) [35], mitochondrial voltage-dependent potassium channel type 1.5 (mitoKv1.5) [36], mitochondrial voltage-dependent potassium channel type 7.4 (mitoKv7.4) [37], large-conductance calcium-regulated potassium channel DEC splice variant (mitoBK_Ca_-DEC) [38], and mitochondrial hyperpolarization-activated cationic channel (mitoHCN) [39]. It is interesting that the above-mentioned channel proteins do not occur simultaneously in the mitochondria, but show tissue specificity. It is documented that “tissue preconditioning” with potassium channel modulators results in cytoprotection through activation of mitochondrial channels [40]. Is not known so far which signaling cascade connects mitochondrial potassium channels with cell protective machinery. It was suggested that the transport of potassium ions into mitochondria via mitochondrial potassium channels causes changes in the volume of the mitochondrial matrix [41, 42], inner membrane potential [43], rate of generation of reactive oxygen species [44], and calcium influx [45, 46]. These changes most likely occur to protect the cell from death [47]. Large conductance calcium-regulated channels are well-described channels in the inner mitochondrial membrane, including in the context of cytoprotection. The mitoBK_Ca_ channel was initially described in 1999 in human glioma cells by Prof. Siemen [33]. Later, it was also identified in the inner mitochondrial membrane of guinea pig ventricular cells [48], skeletal muscles [49], rat astrocytes [50], and neuronal cells [51]. The differences in the regulation of mitochondrial channels by vitamin D offer potential for targeted therapies for diseases such as cancer.

The aim of our research was to elucidate the mechanism by which calcitriol can modulate the mitochondrial potassium channels, as a novel potential non-genomic pathway of vitamin D activity with potential cell-protective properties.

## Methods

### Cell lines

The immortalized human keratinocyte cell line (HaCaT) was obtained from CLS (Cell Line Service, Eppelheim, Germany). U-87MG cells were obtained from ATCC (American Type Culture Collection). Both cell lines were cultured in DMEM medium supplemented with 10% fetal bovine serum, 2 mM L-glutamine, 100 U/ml penicillin, 100 μg/ml streptomycin at 37 °C in a humidified atmosphere with 5% CO_2_.

### Isolation of mitochondria

Mitochondria from cell lines of HaCaT and U-87 MG were isolated using our standard protocol described previously [52, 53]. Briefly, the cells were washed with cold PBS (phosphate-buffered saline), scraped off, and harvested by centrifugation at 800 g for 10 min. After centrifugation, cells were resuspended in isolation buffer (250 mM sucrose, 5 mM HEPES, pH = 7.2) and homogenized using a glass homogenizer. Next, the homogenate was centrifuged at 9200*g* for 10 min to remove cell debris. The supernatant was decanted, and the pellet was suspended in1 ml of preparation solution. The suspension was centrifuged at 770*g* for 10 min. The supernatant was placed in a new tube and centrifuged at 9200*g* for 10 min. Pelleted mitochondria were then resuspended in a storage solution (150 mM KCl, 10 mM HEPES, pH = 7.2) and centrifuged at 9200*g* for 10 min. All steps are under 4 °C.

### Single mitochondria patch-clamp experiments

Mitoplasts (mitochondria without the outer membrane) were prepared from mitochondria from U-87 MG and HaCaT cells by addition of a hypotonic solution (5 mM HEPES, 100 μM CaCl_2_, pH = 7.2) to induce swelling, which is followed by the rupture of the outer membrane. Isotonicity was restored by adding hypertonic solution (750 mM KCl, 30 mM HEPES, 100 μM CaCl_2_, pH = 7.2). Ion current was measured after attaching a free-floating mitoplast to a glass pipette using gentle suction. Mitoplasts were easily recognizable in patch-clamp experiments due to their round shape and presence of a cap (the remnants of the outer membrane) appearing as one or several black spots in phase contrast. Reported voltages are those applied to the patch-clamp pipette interior. Experimental inside-out configuration was proved by observations with various mitochondrial modulators [53]. These modulators, when given in the perfusion pipette, acted on the channels, whereas on the measuring pipette side they did not work.The electrical connection was made using Ag/AgCl electrodes and an agar salt bridge (3 M KCl) as the ground electrode. The current was recorded using a patch-clamp amplifier (Axopatch 200B, Molecular Devices Corporation, USA). The pipettes, made of borosilicate glass, were pulled using a Flaming/Brown puller. The currents were low-pass filtered at 1 kHz and sampled at a frequency of about 100 kHz. Data were analyzed using the pClamp10 software package (Molecular Devices). The open probability of the channel (P_o_) and ion current (I) was determined using the single-channel mode of the Clampfit10 software. To calculate P (open) when more than one channel is present in a patch, the ratio (time open/total time) was further divided by N, the number of channels in the patch. The data are reported as the mean ± SD. Student's t-test was used to evaluate the significance of differences between the two groups.

### RNA extraction, reverse transcription, and real-time PCR experiments

Following 24 h of incubation with vitamin D compounds at 100 nM concentration, total RNA from the keratinocytes was extracted using the ExtractMe total RNA kit (Blirt, Poland), according to the manufacturer’s instructions. The RNA concentrations were determined using an Epoch Microplate Spectrophotometer (BioTek, USA). Extracted RNA was reverse transcribed and cDNA synthesized using a RevertAid First Strand cDNA Synthesis kit (ThermoFisher, USA). Real-Time PCR was performed using a StepOnePlus Real-Time PCR System (Life Technologies Applied Biosystems, Grand Island, NY, USA) with an AMPLIFYME SG No-ROX Mix kit (Blirt, Poland). All primers were purchased from Merck, Munich, Germany. PCR was conducted with the following primers (5ʹ-3ʹ): *KCNN1* forward TCTGCGAGAGGTACCACGA, reverse ATGTCGCCGTAGCCAATGG; *KCNN2* forward AGGAAGCTAGAACTTACCAAAGCA, reverse CCCTGAGTACATTGGCAGCT; *KCNN3* forward TTGACCATGCCAAAGTGAGGA, reverse GCTTGGTCACTCAGCTTCCT; *KCNN4* forward ATGTGGGGCAAGATCGTCTG, reverse CACGTGCTTCTCTGCCTTGT; *KCNMA1* forward CCCGCAGACACTGGCCAATAG, reverse GAGCATCTCTCAGCCGGTAA; *KCNK9* forward CGGCTCCTTCTACTTTGCGA, reverse ACGGCGTAGAACATGCAGAA. The expression of the genes was normalized by the comparative ∆∆-Ct method, using *RPL37A* as a housekeeping gene, followed by calibration (fold change) to normalized expression data of samples from the control (ratio = 1). To ensure the specificity of the PCR amplification, a dynamic melting curve analysis was performed for all reactions.

### Calcitriol preparation and treatment

Calcitriol was purchased from Sigma-Aldrich. Stock solutions of calcitriol were dissolved in ethanol and stored at − 20 °C. Before each experiment, stock samples were diluted in the bath solutions for patch-clamp experiments, or in culture media in the case of qPCR experiments. In our experiments vitamin D derivatives were used at 100 nM concentration, corresponding to the optimal serum 25(OH)D_3_ level (75–125 nM) [54], used in the clinic as a biomarker of vitamin D status [55].

### Molecular docking analyses

Docking analyses of calcitriol to the BK_Ca_ channel were performed using the AutoDock4 program [56]. The crystal structures for the BK_Ca_ channel were taken from the PDB databank. Cryo-EM structure of Ca^2+^-bound hSlo1 channel (PDB: 6V22), and Ca^2+^-free hSlo1 channel (PDB: 6V35) were in resolution 3.2 Å and 3.5 Å respectively. The calcitriol structure was downloaded from the PubChem database and adjusted to PDB format using the PyMOL program. The initial global docking for each channel protein (clustering of 500 structures using root mean square deviation [RMSD] min ligand = 20 Å and making 10 million energy estimates, the parameter was default x = 191.3, y = 191.3, z = 118.4) was performed. The contact between calcitriol and the potassium channel residue was defined when the distance of any two heavy atoms was less than 4 Å. Based on the preliminary results, the search area for calcitriol binding sites was narrowed down to the cytoplasmic domain of the channel. For this purpose, three regions were selected in the cytoplasmic domain. These regions were respectively: the channel pore; the cytoplasmic part of one of the tetramer subunits; and the part between the tetramer subunits, also within the cytoplasmic domain. The search spaces were defined with the parameters size_x = 126; size_y = 126; size_z = 126 (GA runs 400, other parameters were default). All the results obtained were visualized using the PyMOL program.

### Predicting and analyzing transcription factor binding sites

To predict potential transcription factor binding sites within the potassium channel sequence 10 kb upstream of ATG, we used the CiiiDER program from the Hudson Institute of Medical Research [57]. All the sequences were downloaded from the Ensembl website and scanned for transcription factor binding sites using all supplied JASPAR position frequency matrices. In the next step, the binding sites were analyzed taking into account the matrix match score and positions in the tested sequence.

### Statistical analysis

Statistical analysis was performed using Microsoft Excel 2007 (Microsoft Corporation, Redmond, WA, USA) or GraphPad Prism version 6.03 (GraphPad Software, Inc., La Jolla, CA, USA). Data are presented as mean ± SD and were analyzed with Student's t-test (for two groups) or one-way analysis of variance (ANOVA) with appropriate post-hoc tests (for more than two groups) using Prism software. Statistically significant differences are denoted with asterisks: *P < 0.05, **P < 0.01, ***P < 0.005, ****P < 0.0001.

## Results

### Characterization of the potassium ion channels in mitochondria isolated from glioblastoma (U-87 MG) and keratinocyte (HaCaT) cell lines

**Fig. 1:**
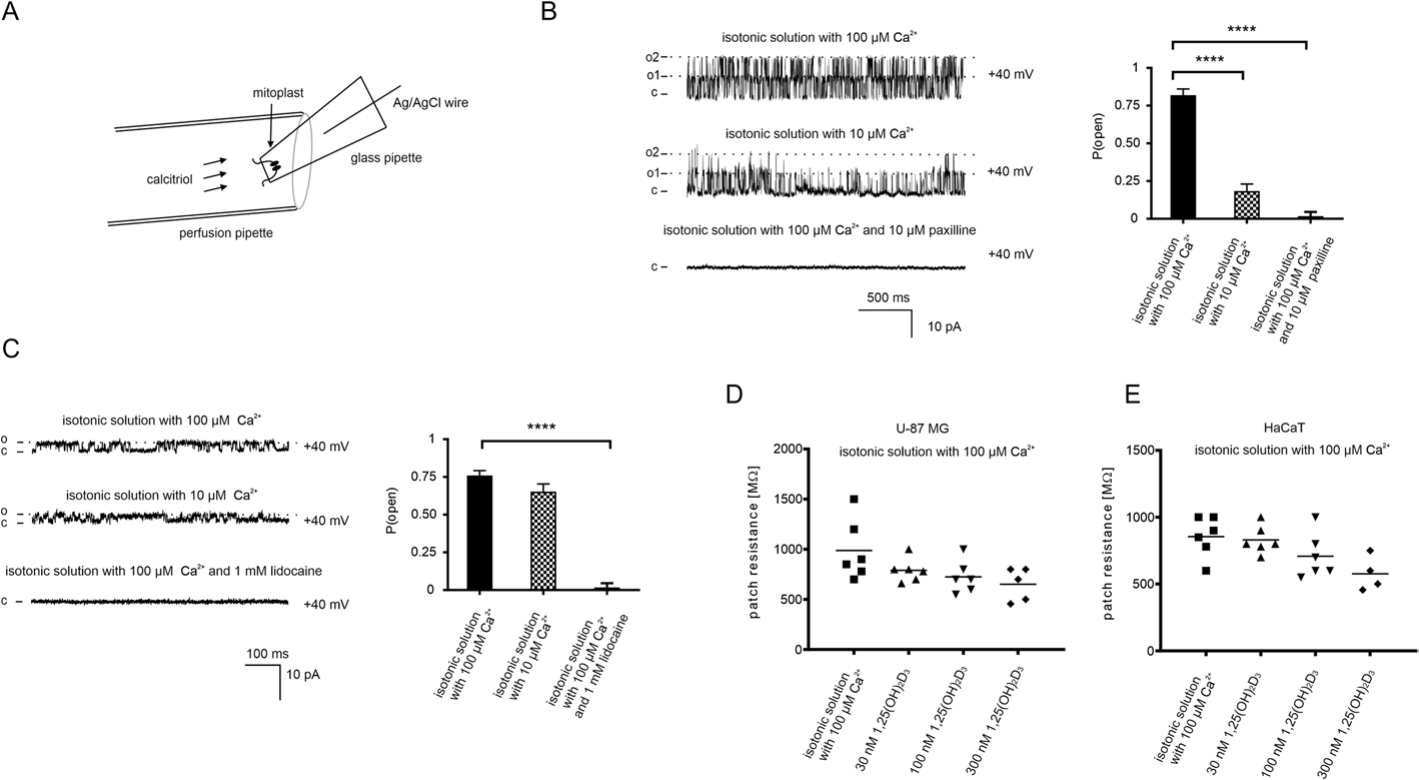
Electrophysiological properties of two potassium channels in mitoplasts isolated from glioblastoma and keratinocyte cell line. **A** Scheme of the patch-clamp experiments performed with calcitriol. **B** Effect of changed calcium ion concentration and paxilline on the activity of the mitoBK_Ca_ channel. (o1) (o2) indicates an open state of channels 1 and 2 respectively. (c) indicates a closed state. **C** Effect of changed calcium concentration and lidocaine on the activity of the TASK-3 channel. (o) indicates an open state of the channel. (c) indicates a closed state. **D**, **E** Resistance of the patch during the external perfusion with calcitriol on mitoplasts from glioblastoma and keratinocyte cells respectively

We decided to study the effect of calcitriol on the activity of well-described mitochondrial potassium channels in two different cell lines due to their selective expression in those cells. As reported previously, in mitochondria from U-87 MG we observed a large-conductance calcium-regulated potassium channel (mitoBK_Ca_), and in mitochondria from HaCaT cells we observed a two-pore domain potassium channel (mitoTASK-3). To confirm the above-mentioned channels, in the first part of our experiments, we characterized them in terms of electrophysiology and pharmacology. The scheme of the patch-clamp experiment and the location of the patch pipette with the attached mitoplast inside the pipe of the perfusion system are shown in Fig. 1A.

The potassium current from mitoplasts isolated from U-87 MG cells was measured in the inside-out mode in a symmetric 150/150 mM KCl isotonic solution in the presence of 100 µM CaCl_2_ (Fig. 1A). To obtain the current–voltage relationship for the channel conductance calculations, channel current traces were recorded at different voltages from + 60 to  − 60 mV. The current–voltage characteristic was linear, current rectification was not observed, and single-channel conductance was equal to 277 ± 10 pS. The open probability was found to be voltage-dependent in a symmetric solution. At negative voltages, the open probability was lower than at positive voltages. Next, for the description of the channel pharmacology, all the recordings were made at the holding potential + 40 mV with tested substances. To test the Ca^2+^ sensitivity of the channel, we reduced the Ca^2+^ concentration from 100 to 10 µM. Recordings demonstrate channel regulation by the low Ca^2+^ concentration and the analysis of P_o_ revealed that the inhibition was statistically significant (Fig. 1B). To exclude the possibility that the measured current was caused by another type of mitochondrial potassium channel, we used the inhibitor paxilline, a substance isolated from *Penicillium* *paxillin*, which is commonly known to block mitoBK_Ca_. Figure 1B presents an example of a single-channel recording measured under control conditions (150/150 mM KCl) and after the addition of 10 μM paxilline. The activity of the channel was irreversibly blocked—this effect is a typical observation for BK_Ca_ channels. Paxilline served also as a control after administration of the test compound.

We conducted similar experiments in the case of potassium currents recorded in mitoplasts isolated from the HaCaT cell line (Fig. 1C). As we described previously [34], in symmetrical conditions the current–voltage characteristic was non-linear, and current rectification was observed. The channel conductance was equal to 77 ± 7 pS at positive voltages and 10 ± 1 pS at negative voltages. Ca^2+^ sensitivity was not observed after the Ca^2+^ concentration was reduced from 100 to 10 µM. Additionally, the current amplitude and P_o_ were not changed in low calcium conditions. To validate the channel during our experiments with the test compound we used the local anesthetic lidocaine. Figure 1C shows that the current amplitude in isotonic solution with 1 mM lidocaine was zero, which indicates that the channel activity was inhibited.

The above-mentioned channels were chosen on purpose to investigate the effect of calcitriol on the potassium channels of the mitochondria. Isotonic solution with calcitriol was added using a perfusion pipette (Fig. 1A). First, we determined the stability of the patches after perfusion of the calcitriol. Figure 1D, E shows electrical resistance of the mitochondrial membrane during the calcitriol application on patch-clamped mitoplasts isolated from glioblastoma and keratinocyte cells. Increased concentrations of calcitriol did not significantly change patch resistance in those two cases. Because our initial experiments showed quite a fast calcitriol response we conducted our experiments in approximately 10-min blocks: control recordings; application of calcitriol; control recordings; application of mitoBK_Ca_ blocker or a TASK-3 channel blocker.

### Calcitriol treatment of the mitochondrial potassium channel from glioblastoma and keratinocyte cells

**Fig. 2:**
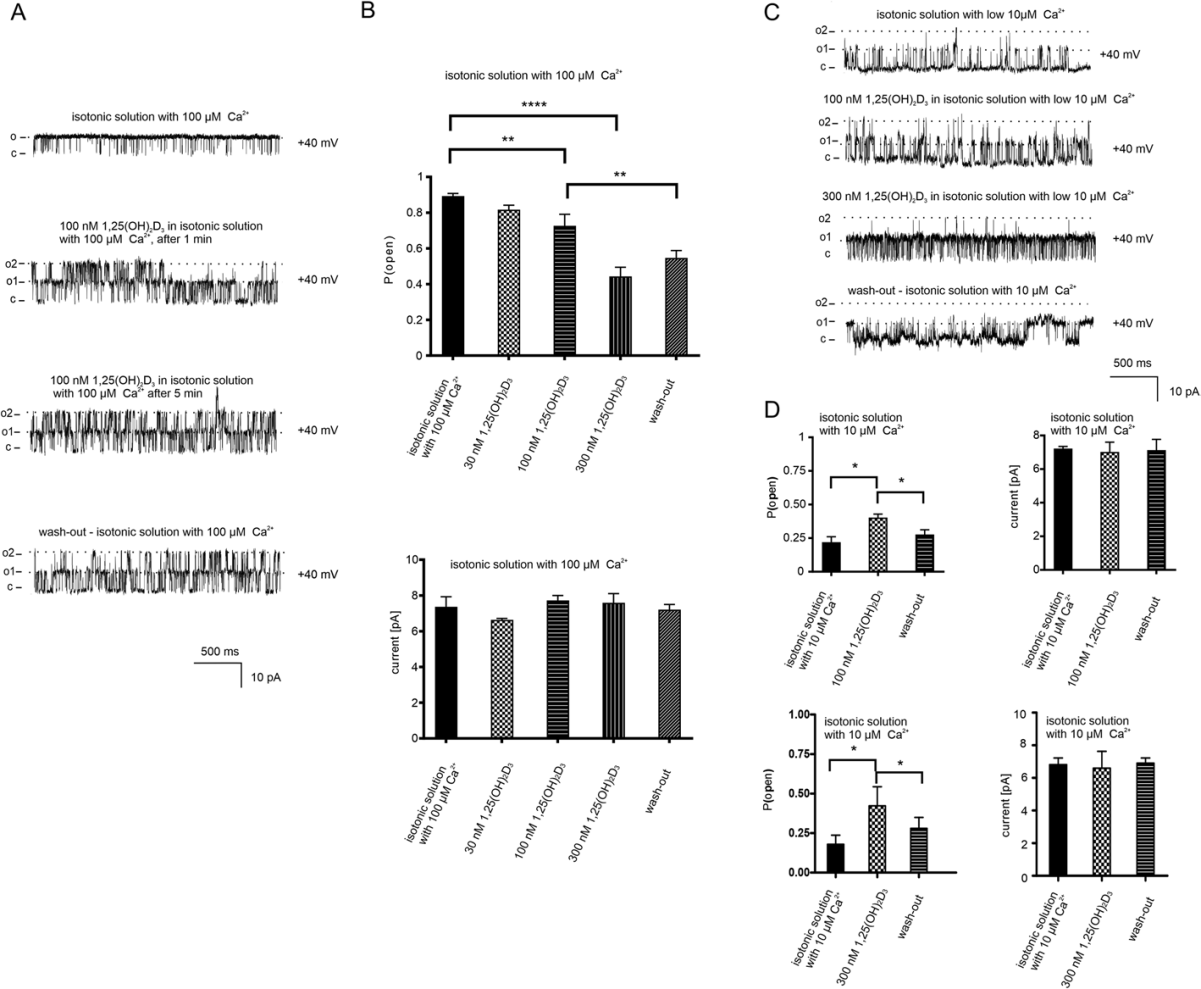
Effect of calcitriol on large-conductance calcium-activated potassium channel from mitochondria isolated from human glioblastoma cells. **A** Recordings at + 40 mV in symmetrical (150/150 mM) KCl solutions with high calcium ion concentration (100 μM Ca^2+^); after application of 100 nM calcitriol (1 and 5 min after application), and after wash-out solution with calcitriol. (o1) (o2) indicates an open state of channels 1 and 2 respectively. (c) indicates a closed state. Recordings are from one representative out of three independent experiments. **B** Bar graphs summarizing the effect of calcitriol on the open probability (P_o_) of the mitoBK_Ca_ channel and the current amplitude (pA) in the presence of 100 μM calcium. The upper panel shows changes of the P_o_ after adding increasing concentrations of calcitriol and wash-out (n = 3). The lower panel shows changes of the current after adding increasing concentrations of calcitriol and wash-out (n = 3). **C** Recordings at + 40 mV in symmetrical (150/150 mM) KCl solutions with low calcium ion concentration (10 μM Ca^2+^, control), after application of 100 nM and 300 nM calcitriol and wash-out. (o1) (o2) indicates an open state of channels 1 and 2 respectively. (c) indicates a closed state. Recordings are from one representative out of three independent experiments. **D** Bar graphs summarizing the effect of 100 nM and 300 nM calcitriol and wash-out on the open probability (P_o_) of the mitoBK_Ca_ channel and the current amplitude (pA) in 10 μM Ca^2+^ ion concentration. The left panel shows changes of the P_o_ after adding 100 nM or 300 nM calcitriol and wash-out (n = 3). The right panel shows changes in the current after adding 100 nM or 300 nM calcitriol and wash-out (n = 3)

After characterizing the mitoBK_Ca_ properties in U-87 MG cells, the effect of calcitriol on the single channels in opposite conditions was studied: (1) when the open probability of the channel is high (in isotonic solution with 100 μM Ca^2+^), and (2) when the open probability of the channel is low (in isotonic solution with 10 μM Ca^2+^). The effect of three different concentrations of calcitriol was tested: 30 nM, 100 nM, and 300 nM. In a 100 μM Ca^2+^ isotonic solution, only applications of 100 nM and 300 nM significantly change the open probability of the channel in both conditions. Figure 2A shows channel recordings at + 40 mV in control conditions and after addition of 100 nM calcitriol during 1 min, and 5 min after application. The strongest effect of calcitriol was observed after 5 min and was slightly reversible. After calcitriol treatment, the open probability of the channel from one to five minutes decreased linearly, was the lowest after five minutes, and reached a plateau. Taking into account such observations, all the calculations were performed after 5 min of the calcitriol treatment. Figure 2B summarizes the effect of calcitriol on the open probability and current amplitude of the mitoBK_Ca_ channel at different calcitriol concentrations. Analogous experiments were carried out in low calcium concentration conditions (10 μM Ca^2+^). Figure 2C shows representative current traces of the mitoBK_Ca_ channel in isotonic solution at + 40 mV, after 100 nM and 300 nM calcitriol and wash-out. Similarly, 100 nM and 300 nM calcitriol affect the channel. The open probability of the channel increased but the current amplitude did not change significantly. The effect of calcitriol was reversible (Fig. 2D).

**Fig. 3:**
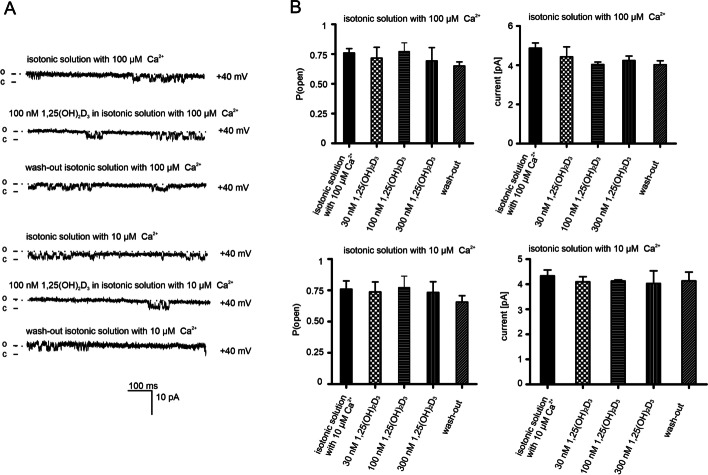
Effect of calcitriol on two-pore domain potassium channels from mitochondria isolated from human keratinocytes. **A** Recordings at + 40 mV in symmetrical (150/150 mM) KCl solutions with high calcium ion concentration (100 μM Ca^2+^, control) and low calcium concentration (10 μM Ca^2+^, control) and after application of 100 nM calcitriol. (o) indicates an open state of the channel. (c) indicates a closed state. Recordings are from one representative out of three independent experiments. **B** Bar graphs summarizing the effect of calcitriol on the open probability (P_o_) of the mitoTASK-3 channel and current amplitude (pA) in the presence of high and low calcium conditions. The left panel shows changes of P_o_ after adding 30 nM, 100 nM, 300 nM calcitriol in the presence of high and low Ca^2+^ ion concentration (n = 3). The right panel shows changes in the current after adding 30 nM, 100 nM, 300 nM calcitriol in the presence of high and low Ca^2+^ ion concentration (n = 3)

To test whether calcitriol affects the calcium-independent mitochondrial potassium channel, mitoplasts isolated from human keratinocytes were used. However, no changes in channel activity were observed after applying 30, 100, and 300 nM calcitriol. Current traces for representative channel recordings at holding potentials of + 40 mV are shown in Fig. 3A. Open probability and current are shown in Fig. 3B.

### Measurements of the expression level of the potassium channels after calcitriol treatment—prediction of VDR binding sites

**Fig. 4:**
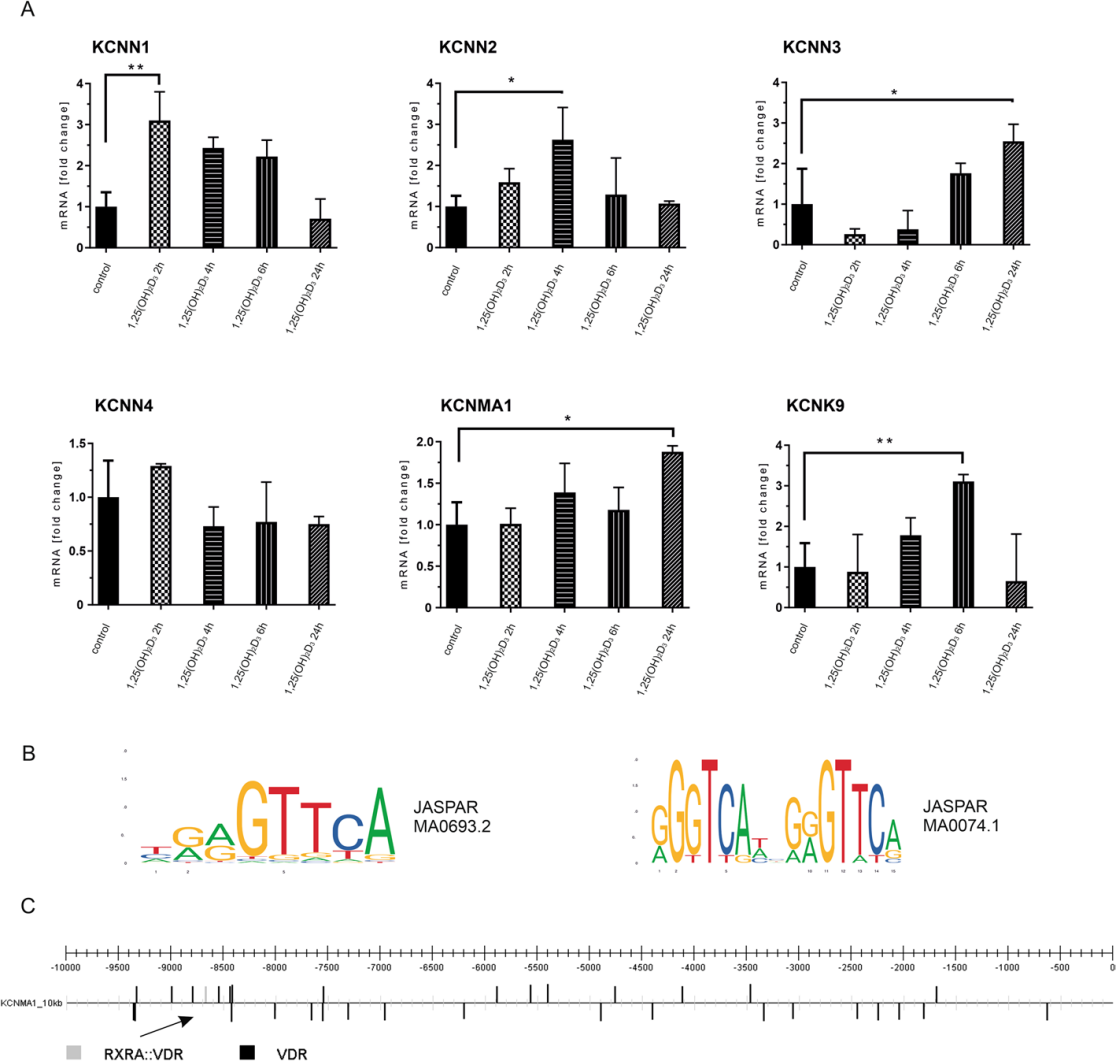
Calcitriol effect on expression level of potassium channels. **A** Calcitriol effect on expression of calcium-regulated potassium channels (*KCNN1-KCNN4; KCNMA1*) and two pore-domain potassium channel TASK-3 (*KCNK9*) in HaCaT cells. **B** JASPAR matrices are used in CiiiDER program analysis. **C** Prediction of VDR binding sites in the BK channel gene (*KCNMA1*) by the CiiiDER program. VDR binding sites (JASPAR matrix MA0693.2) in *KCNMA1* sequence 10 kb upstream of ATG were marked in black. VDR binding site (JASPAR matrix MA0074.1) in *KCNMA1* sequence 10 kb upstream of ATG was marked in grey

To evaluate the possible genomic effect on the expression of potassium channels after calcitriol treatment, we conducted qPCR experiments. For the analysis we chose small-conductance calcium-regulated potassium channels SK1 (gene: *KCNN1*), SK2 (gene: *KCNN2*), SK3 (gene: *KCNN3*), intermediate-conductance calcium-regulated potassium channel IK (gene: *KCNN4*), large-conductance calcium-regulated potassium channel BK_Ca_ (gene: *KCNMA1*) and two-pore domain potassium channel TASK-3 (gene: *KCNK9*) (Fig. 4A). After two or four hours of calcitriol treatment, we observed up-regulation of the *KCNN1* and *KCNN2* gene expression levels. After four or six hours, we observed an increase of expression in the case of *KCNN3*, *KCNMA1*, and *KCNK9*. Expression of the *KCNN4* gene did not change significantly after adding calcitriol. So far, there are no reliable antibodies to detect the studied channels available, which prevent us from conducting analyses of protein products.

Thus, after the qPCR experiments, an in silico analysis was conducted to predict putative VDR binding sites in potassium channel promoter sequences. For this purpose, we used the CiiiDER program [57]. Sequences of potassium channels of interest, about 10 kbp upstream of ATG, were downloaded from the Ensembl website. Matrices for VDR (MA0693.2), VDR:RXR (MA0074.1), CEBPA (MA0102.4), and CTCF (MA0139.1) were downloaded from the JASPAR database (Fig. 4B). Figure 4C shows the VDR binding sites in the sequences upstream of ATG of the selected potassium ion channel genes. In the *KCNMA1* sequence, we found 34 sites for VDR, and one site for RXRA:VDR (all the matrix match scores were between 0.8 and 1). In *KCNK9* 21 sites for VDR; in *KCNN1* 27 VDR sites; in *KCNN2* 35 sites for VDR; in *KCNN3* 39 VDR sites. Interestingly, one DR-3 VDR:RXR binding sequence was found in the sequence upstream of the BK channel. Additionally, only in the case of the *KCNMA1* gene, 13 CEBPA transcription factor binding sites were found with the matrix match score between 0.85 and 0.97. CEBPA (CCAAT/enhancer-binding protein) is a leucine zipper transcription factor abundantly expressed in keratinocytes of the skin. CEBPA helps other settler transcription factors, such as VDR, to bind to their targets within chromatin. In the upstream *KCNMA1*sequence, there were also found two CTCF binding sites with a matrix match score of 0.96 and 0.86. CTCF (CCCTC-binding factor) transcription factor contributes to DNA looping and marks the anchors of 587 putative topologically associating domains containing at least one VDR binding site and one 1,25(OH)_2_D_3_ target gene. These domains can explain the regulatory scenarios of up to 70% of all 1,25(OH)_2_D_3_ target genes. All potential VDR binding sites generated with the CiiiDER program were included in Additional file 1: Fig. S1.

### Molecular docking analysis of calcitriol to the BK channel

**Fig. 5:**
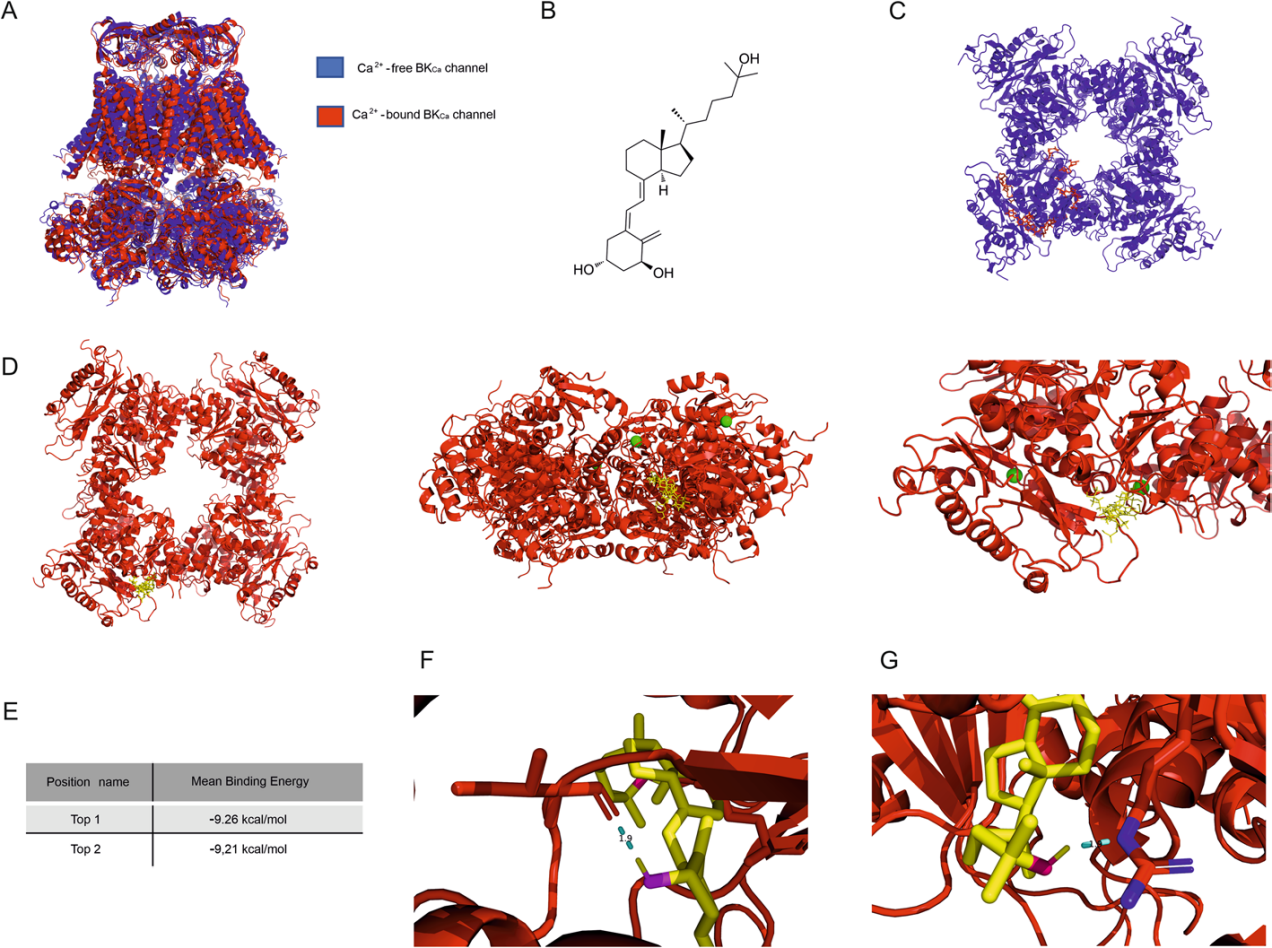
Predicted docking poses of calcitriol on the BK_Ca_ channel with and without Ca^2+^and a close-up of the specific residues predicted to interact with calcitriol. **A** Protein alignments of two structures in 3D, BK_Ca_ channel Ca^2+^-bound (red color; PDB: 6v22) and BK_Ca_ channel Ca^2+^-free (blue color; PDB: 6v35). **B** calcitriol molecule **C** Docking poses of calcitriol (red color) on the cytoplasmic domain of the Ca^2+^-free BK_Ca_ channel, showing wide diversity of binding poses. **D** Predicted docking poses of calcitriol (yellow color) on the cytoplasmic domain of the Ca^2+^-bound (green spheres) BK_Ca_ channel. **E** The predicted binding energies of two calcitriol binding poses (top 1, top 2). **F** Close-up of the specific residues of the BK_Ca_ channel protein predicted to interact with calcitriol (top 1) hydroxyl group on C1 calcitriol carbon. **G** Close-up of the specific residues of the BK_Ca_ channel protein predicted to interact with calcitriol (top 1) hydroxyl group on C25 calcitriol carbon

To investigate the potential sites of interaction of the calcitriol molecule with the mitoBK_Ca_ channel, we undertook a computational analysis. Since no mitochondrial BK_Ca_ channel crystal structure is available, analyses were carried out on the solved plasma membrane channel structures. We chose the cryo-EM structure of the Ca^2+^-bound hSlo1- BK_Ca_ channel (PDB: 6V22) and the Ca^2+^-free hSlo1- BK_Ca_ channel (PDB: 6V35). Based on the available knowledge, the cytoplasmic domain of the BK_Ca_ channel should most likely be the binding site for calcitriol (Fig. 5A, B). The cytoplasmic domain was taken into account by us due to the ability of this domain to bind cholesterol. We performed global docking and docking to selected regions of the cytoplasmic domain of the BK_Ca_ channel (bound and not bound to calcium ions). As can be seen in Fig. 5A, the spatial structure of the BK_Ca_ channel after binding calcium ions differs from that not related to calcium ions. In the case of the BK_Ca_ channel not bound to calcium ions, both global and local docking of calcitriol showed a large variety of structures (binding sites), but these bonds were not strong (Fig. 5C). In the case of the Ca^2+^-bound BK_Ca_ channel (Fig. 5D), we found two calcitriol binding sites (top 1 and top 2) with mean binding energies of − 9.26 kcal/mol and − 9.21 kcal/mol respectively (Fig. 5E). It should be emphasized that both global and local ligand docking indicate that the likely site for calcitriol binding is the cytoplasmic domain of the calcium-bound channel. Figure 5F shows the calcitriol binding sites with the marked hydrogen bonds between the hydroxyl group on C1 calcitriol carbon and the carboxyl group of Ile^876^. Figure 5G shows the calcitriol binding sites with the marked hydrogen bonds between the hydroxyl group on C25 calcitriol carbon and the guanidyl group of Arg^1031^. Specifically, two calcitriol binding sites are predicted to interact (share residues) with Gln^501^, Lys^828^, Val^874^, Asn^875^, Ile^876^, Pro^877^, Ile^878^, Thr^913^, Thr^1029^, Lys^1030^, Arg^1031^. Residues Leu^891^, Asp^892^, Tyr^1015^, and Leu^1017^ are unique to top1 (the lowest mean binding energy), while residues Asn^795^, Leu^796^, and Cys^797^ are unique to top 2. Additionally, using the Geneious Prime program, we checked in which domains of the channel protein the above-mentioned amino acids are located. In the case of top 1, Gln^501^ is located in segment S7, Leu^891^ and Asp^892^ are in the calcium-bowl region and Thr^913^ is in segment S10. The other amino acids are located between segments S8 and S9, S9 and S10 of the BK_Ca_ channel cytoplasmic domain.

## Discussion

Steroid hormones (SSH), including calcitriol, can regulate potassium ion channels via direct binding to a channel protein or to an auxiliary protein that regulates the gating properties of the channel. Another possibility is indirect regulation via signaling pathways initiated from SSH receptors in the plasma membrane or cytosol including classic genomic transcriptional pathways or by changing the properties of the lipid bilayer in which the channels are located [58–60]. So far, there is no literature data on the influence of calcitriol on the activity of the potassium channels from the inner mitochondrial membrane, and in the case of the cell membrane, this knowledge is reduced to a few studies. Here, we report for the first time that active forms of vitamin D directly affect the activity of the mitochondrial large-conductance calcium-regulated potassium channel.

Previously it was shown that estradiol binds to the auxiliary beta1 subunit of the plasmalemma BK_Ca_ channel, namely with hydrophobic residues in the second transmembrane domain and activated channel in smooth muscle cells [61]. In the case of the mitochondrial BK_Ca_ channel the effect of estradiol is quite the same; estradiol increased P_o_ of the mitochondrial BK_Ca_ channel in U-87 MG cells during the first minutes after application, and closed the PTP channel, which maybe protective for the cell, by preventing the cell from undergoing apoptosis [62].

Some studies show that the influence of calcitriol on the activity of ion channels is dependent on the VDR receptor. For example, calcitriol increased the L-type calcium current and the fast transient outward current K^+^ current in mouse ventricular myocytes, and the effect was absent in myocytes isolated from VDR knockout mice [63, 64]. We did not observe VDR immunoreactivity in the mitochondria of HaCaT keratinocytes (not shown), but activity characteristic for BK_Ca_was also not detected in those cells; thus the influence of VDR may not be excluded.

As yet, no electrophysiological data are available on the regulation of the mitochondrial and plasma membrane BK_Ca_ channel by calcitriol. In one study, it was speculated that the structural similarity of vitamin D to cholate lithocholic acid, which activates BK_Ca_ channels, makes BK_Ca_ channel activation highly probable under the influence of vitamin D. In the case of the widely studied cholesterol, both direct and indirect forms of regulation have been described [56, 65, 66]*.* The experimental analysis showed that cholesterol can directly interact with the channel (cytoplasmic domain) [66]. Cholesterol also activates the BK_Ca_ channel with the beta1 auxiliary subunit. Moreover, cholesterol regulation, from inhibition to activation, is determined by a trafficking-dependent increase in membrane levels of beta1 subunits in smooth muscle cells [67].

In our experiments, calcitriol lowered or increased the probability of opening the mitochondrial BK_Ca_ channel depending on calcium ion concentration. Our electrophysiological experiments were carried out in patch-clamp inside-out mode, which means that calcitriol was applied from the mitochondrial matrix site. Additionally, this was confirmed by our experiments using calcium ions. Calcium ions immediately affect the probability of channel opening when administered from the side of the perfusion pipette. It was proven that the calcium sensor in the BK_Ca_ channel structure is a large tetrameric structure called the “gating ring” [68]. Each subunit of this structure contains two distinct regulators of conductance of potassium domains, each contributing one Ca^2+^ binding site [69]. To evaluate possible calcitriol binding to the BK_Ca_ channel we conducted in silico simulations using the AutoDock4 program. As our experimental data indicated differences in channel responses to calcium ion-dependent calcitriol modulation, we analyzed the potential binding site of calcitriol to the solved crystallographic structure of the channel bound and not bound to calcium ions. For our docking simulation, we chose BK_Ca_ channel crystal structures regulated by the beta4 auxiliary subunit. From the previous experiments on the U-87 MG cells, we know that the mitochondrial BK_Ca_ channel can interact with beta4, and such subunit was proved to be localized in mitochondria [53]. Comparing the results of electrophysiological and docking experiments, we can conclude that calcitriol has a more specific effect on the BK_Ca_ channel associated with calcium ions than on the calcium-free channel. Both global and local ligand docking indicates that the likely site for calcitriol binding is the cytoplasmic domain of the calcium-bound channel. Analyses of mitochondrial channel topology indicate that this domain is present in the mitochondrial matrix. Therefore, under physiological conditions, calcitriol would pass through the mitochondrial membranes and bind to the channel from the matrix side. Interestingly, the docking results for top1 and top2 show that contact amino acids (Leu^891^ and Asp^892^) are present in the calcium-bowl region. Analyzing the results of experiments under low calcium conditions, we can conclude that calcitriol does not bind strongly to the channel. However, we must not forget that in electrophysiological experiments we observed a slight increase in the probability of channel opening after administration of calcitriol, and this effect was reversible. Comparing the binding sites of calcitriol with cholesterol, we can conclude that the only site common to both ligands is the cholesterol-binding motif (CRAC1), including the amino acids between 1010 and 1018 sites [66].

It should be emphasized that all measurements made by us were in the range of − 60 to + 60 mV. Under physiological conditions, the inner mitochondrial membrane potential is about − 140 mV. However, we are unable to measure channels under such conditions due to membrane instability resulting in patch detachment. Under physiological conditions the mitochondrial BK channel activity is low (the open probability of the channel at − 140 mV is low), but when the calcium ions are in the mitochondrial matrix relationship open probability vs. potential moves to the left side to lower potentials.

Although our results are more indicative of a direct effect of calcitriol on the channel, we cannot completely exclude the effect of calcitriol on the cell membrane, whose changing properties also affect the channel itself. Most research on the influence of calcitriol on the physicochemical properties of membranes is from the 1980s. Administration of 1,25(OH)_2_D_3_ leads to an increase in the de novo synthesis of phosphatidylcholine (PC) and an increase in the total PC content. It also increases the turnover of fatty acids into PC, which increases the content of polyunsaturated fatty acids in the PC fraction. 1,25(OH)_2_D_3_ regulates PC membrane content, increases membrane fluidity, and increases the rate of calcium transport through the calcium channel regulated by calcitriol [70]. Membrane fluidity measured by fluorescence anisotropy of 1,6-diphenyl-1,3,5-hexatriene was significantly greater in rat intestinal brush border membranes treated with vitamin D_3_ [71]. In chondrocytes calcitriol action results in rapid changes in the arachidonic acid release and re-incorporation, alterations in membrane fluidity and Ca^2+^ flux [72]. Other data suggest that vitamin D specifically modulates fatty acid composition in adipose tissue through direct regulation of Elovl3, an enzyme that functions in the synthesis of C20–C24 saturated and mono-unsaturated long-chain fatty acids [73]. In the case of mitochondrial membrane, there are some reports that the fluorescence anisotropy in the mitochondria from vitamin D-treated chicks is significantly lower than that from the vitamin D-deficient animals. The fluorescence studies performed in mitochondrial subfractions revealed that cholecalciferol treatment induces a decrease of the lipid order parameter in the mitochondrial inner membrane [74]. In our case, the influence of calcitriol on the inner mitochondrial membrane cannot be ruled out, but we can conclude that the patch resistance did not change suddenly after administration of the ligand and that patches remained during the measurement. In addition, in the Geneious program, we checked that the sites of ligand contact with the protein are not present in the STREX motif, which is responsible for the mechanosensitivity of the channel.

The genomic effect of calcitriol on potassium channels was also analyzed. For this purpose, we chose not only the BK_Ca_ channel and TASK-3, but also other potassium ion channels regulated by calcium ions (including IK, SK1, SK2, SK3). In general, after short stimulation (2 or 4 hours), we observed an increase in the expression of the *KCNN1* and *KCNN2* genes. In the case of longer stimulations, the increase in expression was visible for the *KCNN3, KCNMA1*, and *KCNK9* genes. Our analysis of the promoter sequences of the studied genes using the CiiiDER program indicated a high probability of a VDR-dependent genomic effect. Nevertheless, the analyses carried out were theoretical and require further extensive experimental research. It should be noted, however, that the current published works, although they are few, generally indicate a decrease in the expression of genes encoding selected potassium channels by calcitriol. In the case of the BK_Ca_ channel (*KCNMA1*) from breast cancer cells, real-time PCR assay showed that expression levels of the channel transcripts treated with 1 µM calcitriol for 72 h were more than 90% lower than in the control [75]. A similar result was obtained in the case of EAG1 gene expression. ERG channels were down-regulated by calcitriol in cancer cells from the cervix, prostate, and mammary gland [28]. This inhibition was also observed in non-tumor cells, namely, syncytiotrophoblasts, primary cultures from normal term placenta. Interestingly, calcitriol had an effect on EAG1 mRNA. Calcitriol exerted stronger inhibition on EAG1 gene expression in cells overexpressing VDR. It was suggested that such mRNA repression by calcitriol involves a functional negative vitamin response element (nVDRE) E-box type in the hEAG1 promoter [76].

Summarizing the above considerations, in astrocytoma cells, regulation of the mitochondrial BK_Ca_ channel by calcitriol is calcium-dependent. According to the literature, in general, the opening of some of the mitochondrial potassium channels may lead to cell protection, and the closing of the channels may lead to apoptotic events [77]. It was also proved that an increase in the activity of the mitoBK_Ca_ channel in the inner mitochondrial membrane reduces the activity of the permeability transition pore (PTP) [50]. Also, it should be mentioned that the respiratory chain lowers the P_o_ of the mitoBK_Ca_ channel in the presence of substrates [53]. The higher expression of the BK_Ca_ channel gene after calcitriol treatment observed by us does not necessarily result in high channel activity, and the channel protein can be present in mitochondrial and/or plasma membrane. Maybe in a long run, genomic activity of calcitriol helps mitochondria in the regulation of ion homeostasis, by increasing the channel number, but the rapid non-genomic response implies direct calcium-dependent interaction with the mitoBK_Ca_ channel. However, at this stage of the work, we must be very careful in drawing premature conclusions.

## Conclusions

In summary, the main findings of the present study are as follows: (1) calcitriol regulates the open probability of the mitochondrial BK_Ca_ channel depending on calcium level in the U-87 MG cell line; (2) calcitriol did not change the open probability or current of the TASK-3 channel from the keratinocyte cell line; (3) the expression level of the potassium channel genes is changed after calcitriol treatment; (4) calcitriol can be bound to the cytoplasmic domain of the calcium-bound BK_Ca_ channel more specifically than to the calcium-free BK_Ca_ channel cytoplasmic domain.

Taken together, our results provide direct evidence for a novel link between calcitriol and mitochondrial bioenergetics. It should also be emphasized here that the specificity of calcitriol to a given potassium channel from the mitochondria, and consequently from the specific tissue, opens up new therapeutic possibilities of calcitriol.

## Supplementary Information

**Additional file 1: Figure 1.** VDR, VDR:RXR, CEBPA, CTCF binding sites analyzed by the Ciiider program in case of mitochondrial potassium channels.

## Data Availability

The authors declare that all data generated or analyzed during this study are included in this published article and its additional information files.

## Abbreviations

ATP5B: Mitochondrial membrane ATP synthase subunit beta
BK_Ca_: Large-conductance calcium-regulated potassium channel
BK_Ca_-DEC: Large-conductance calcium-regulated potassium channel DEC splice variant
CEBPA: CCAAT enhancer binding protein alpha
COX 2, 4: Cyclooxygenase-2, -4
CRAC: Cholesterol-binding motifs
CTCF: CCCTC-binding factor
CYP24A1: Cytochrome P450 family 24 subfamily A member 1
CYP27A1: Cytochrome P450 family 27 subfamily s A member 1
CYP27B1: Cytochrome P450 family 27 subfamily B member 1
EAG: Voltage-gated ether à go-go potassium channel
ERp57: Endoplasmic reticulum resident protein 57
HaCaT: Human keratinocyte cell line
IK: Intermediate-conductance calcium-regulated potassium channel
KCNK9: Two-pore domain channel type 3 channel gene
KCNMA1: BK_Ca_ channel gene
KCNN1: Potassium calcium-activated channel subfamily N member 1 gene
KCNN2: Potassium calcium-activated channel subfamily N member 2 gene
KCNN3: Potassium calcium-activated channel subfamily N member 3 gene
KCNN4: Potassium calcium-activated channel subfamily N member 4 gene
MARRS: Membrane-associated rapid response to steroid
mitoBK_Ca_: Mitochondrial large-conductance calcium-regulated potassium channel
mitoHCN: Mitochondrial hyperpolarization-activated cationic channel
mitoIK: Mitochondrial intermediate-conductance calcium-regulated potassium channel
mitoKv1.3: Mitochondrial voltage-dependent potassium channel type 1.3
mitoKv1.5: Mitochondrial voltage-dependent potassium channel type 1.5
mitoKv7.4: Mitochondrial voltage-dependent potassium channel type 7.4
mitoSK: Mitochondrial small-conductance calcium-regulated potassium channel
mitoTASK-1: Two-pore domain channel type 1 channel
mitoTASK-3: Two-pore domain channel type 3 channel
MT-ATP6: Mitochondrially encoded ATP synthase membrane subunit 6
PC: Phosphatidylcholine
P_o_: Open probability of the channel
PTP: Permeability transition pore
ROS: Reactive oxygen species
SSH: Steroid hormones
StAR: Steroidogenic acute regulatory protein
TASK-1: Two-pore domain type 1 channel
TASK-3: Two-pore domain type 3 channel
TCA: Tricarboxylic acid cycle
TOM/TIM: Translocase of the outer and inner mitochondrial membrane
TRPV: Transient receptor potential cation channels
U-87 MG: Human astrocytoma cell line
VDAC: Voltage-dependent anion-selective channel
VDR: Vitamin D receptor
VDRE: Vitamin D response element
